# Supplementary material for: Comfort or conservation? Investigating patient choices between plastic and metal speculums
Source: PLoS One. 2026 Apr 15;21(4):e0346819. doi: 10.1371/journal.pone.0346819 (PMC13082593; doi:10.1371/journal.pone.0346819)
Supplement: Attachment — Submitted filename: Letter of Revisions.docx [file pone.0346819.s003.docx]

Dear Reviewers,

We have addressed all the issues noted in the revision suggestions. I have noted the changes based on the line numbers of the new document rather than the old as many things have shifted lines.

Line 20 shows the corrected discrepancy between the IRB dates and recruitment period start date. We apologize for the confusion.

Line 34-35 (previously lines 33-34) was revised to avoid grammatically award phrasing.

Line 49 (previously line 47) was revised for its inconsistent superscript placement.

Lines 62-66 & Lines 71-89 use the new reference that replaced the old reference 8 that was not a peer reviewed research article. After finding appropriate references, more lines were added in for context as they added more to our paper. In addition, we removed and replaced two other website sources (old references 10&11) that were not peer reviewed articles with an appropriate reference (new reference 8) that also added more to the paper.

Lines 120-123 & Lines 218-219 address the issue of the lack of power calculation and those implications with the potential of being underpowered to detect small subgroup differences.

Lines 125-129 address survey instrument validation, piloting, and whether there were previously validated instruments for addressing this topic.

Lines 132-135 addressed the response rate and how many patients were approached and did not complete the survey. Potential selection bias has been reported in this section.

Lines 147-150 address the lack of formal correction for multiple comparisons following the chi-square results and how the findings should be interpreted as hypothesis-generating.

Lines 174-174 provide the chi-square statistic, degrees of freedom, and P-value.

Lines 152-154-149 & Line 200 below Table 1 address the fact that some percentages may not total to 100% due to rounding.

Lines 163-167 address how incomplete or missing data was handled as well as how many surveys were submitting in full versus partially complete and how many were used for the primary outcome of this paper.

Lines 223-229 address the lack of collection of demographics in patients as well as the predominantly non-Hispanic White population of West Virginia. This source is straight from the U.S. Census Bureau. If it would be preferred for a peer-reviewed article to be used, that can be addressed.

Lines 280-2292 rephrases the speculative statement regarding environmental concerns affecting speculum preferences. Clarification that no trade-off decision making between comfort and sustainability was assessed was added as well.

Regarding deciding between using speculums or specula, we decided to be consistent with common usage in biomedical literature that PLOS style suggests in order to emphasize accessible language rather than strict Latin plurals. Therefore, we retained “speculums” throughout the manuscript for consistency instead of “specula.”

Figure 2 has been revised to replace “metallic” with “metal” to stay consistent with the rest of the paper.

Table 2: In regard to more accurately citing the references used for table 2, we found a more accurately applicable source for this table and cited each row by source. Therefore, we edited the table and added in more to the discussion centered around this new reference. The added discussion is seen in lines 295-301.

Lines 339-241 were added to address the data availability. It has been made publicly available via the link provided.

Overall, some relocation of sentences and grammar editions have been implemented. Additionally, the references have been re-ordered based on where they fall in number within the paper. These track changes have been noted but not pointed out in this document as they are ultimately the same reference.

Thank you for considering our study and suggesting these helpful revisions. If you have issues accessing the data or have other suggestions, please let us know. We would be happy to revise again.

Sincerely,

Torren Kalaskey
